# An immediate postoperative response to therapy assessment can help avoid unnecessary RAI therapy

**DOI:** 10.3389/fonc.2022.947710

**Published:** 2022-08-12

**Authors:** Hao Zhao, Chun-Hao Liu, Yue Cao, Li-Yang Zhang, Ya Zhao, Xin Zhang, Yan-Song Lin, Yu Xia, Yue-Wu Liu, Hong-Feng Liu, Xiao-Yi Li

**Affiliations:** ^1^ Chinese Academy of Medical Sciences & Peking Union Medical College, Beijing, China; ^2^ Department of General Surgery, Peking Union Medical College Hospital, Chinese Academy of Medical Sciences & Peking Union Medical College, Beijing, China; ^3^ Department of Nuclear Medicine, Peking Union Medical College Hospital, Chinese Academy of Medical Sciences & Peking Union Medical College, Beijing, China; ^4^ Department of Ultrasound, Peking Union Medical College Hospital, Chinese Academy of Medical Sciences & Peking Union Medical College, Beijing, China

**Keywords:** differentiated thyroid cancer, radioiodine therapy, response to therapy, capsular invasion, lymph node metastases

## Abstract

**Background:**

Radioiodine (RAI) therapy plays a vital role in the postoperative treatment of differentiated thyroid cancer (DTC) patients underwent total thyroidectomy (TT). However, even in the presence of capsular invasion and lymph node metastasis prognosis can be excellent and a postoperative RAI treatment might not be necessary for all patients. Therefore, this study explored the criteria for avoiding unnecessary RAI therapy in these patients.

**Method:**

We applied response to therapy assessment immediately after surgery and prospectively recruited 179 excellent or indeterminate response DTC patients with capsular invasion and/or LNM who underwent TT without RAI therapy. During the follow-up, thyroglobulin (Tg), thyroglobulin antibody (TgAb) levels, and cervical ultrasonography were collected and analyzed. Disease-free survival (DFS) was calculated using the Kaplan-Meier method. In addition, response to therapy assessments was performed on patients during each follow-up.

**Results:**

The mean follow-up period was 29.85 ± 17.44 months, and the 3- and 5-year DFS for all the patients was 99.3% in each. At the last follow-up, 165 (92.2%) patients had excellent responses, while 12 (6.7%) had an indeterminate response, and one (0.6%) each had biochemical and incomplete responses. No significant difference was observed in response to therapy between the subgroups of LNM and tumor invasion (P>0.05). For patients with capsular invasion and a number of metastatic lymph nodes ≤5 and >5, the proportions of recorded excellent responses were 95.9%, 91.0%, and 85.7%, respectively. Better responses were observed in females (excellent response: 95.5%, P=0.023), patients with stimulated Tg (s-Tg) ≤1ng/ml (excellent response: 100%, P<0.001), s-Tg ≤ 2ng/ml (excellent response: 98.4%, P<0.001), and excellent response for the immediate postoperative assessment (excellent response: 98.5%, P=0.004).

**Conclusions:**

The current study suggested that the response to therapy assessment immediately applied postoperatively could help avoid unnecessary RAI therapy among DTC patients with capsular invasion and/or LNM. Moreover, excellent response patients and patients with indeterminate response and s-Tg ≤ 2ng/ml could be managed without RAI therapy.

## Introduction

Differentiated thyroid cancer (DTC) is the most common thyroid malignancy, accounting for 85-90% of thyroid cancer (1, 2). The standard treatments of DTC include surgical resection, radioactive iodine (RAI) therapy, and thyroid-stimulating hormone (TSH) suppression (3). Therefore, the decision to perform RAI therapy is an essential consideration after total thyroidectomy (TT). Generally, RAI therapy is unnecessary for low-risk thyroid microcarcinomas (TMC). In contrast, it is routinely undergone in high-risk patients with gross extrathyroidal invasion or distant metastases (DM). However, RAI therapy in patients with risk of recurrences, such as capsular invasion and lymph node metastases (LNM), remains controversial (4). Thus, the benefits of RAI therapy remain uncertain in these patients. Furthermore, it may cause adverse effects like sialadenitis and rise the concern of second primary malignancy (SPM) (5–9). Therefore, the pros and cons of RAI therapy should be weighed in these patients.

Previous studies have reported that capsular invasion and LNM occurred in 38.1%-49.8% and 25.7%-60% of the overall DTC patients, respectively, indicating a large amount of patients with uncertain RAI decisions (10–12). Several studies have revealed that RAI treatment improves overall survival (OS) and disease-free survival (DFS), especially in patients with the involvement of multiple lymph nodes (LN) (13–15). However, some studies contradicted such outcomes. For example, a study by Ballal et al. with a median follow-up of 10.3 years indicated no significant difference in the overall prognosis of intermediate-risk patients undergoing RAI therapy (16). Similarly, in a large sample study involving 8297 intermediate-risk patients, Kim et al. reported that RAI therapy could not reduce the local recurrence risk (LRR, hazard ratio (HR) = 0.852, P = 0.413) (17). Thus, a further investigation into whether patients with recurrence risk can benefit from RAI therapy and how the treatment should be administered is warranted.

Response to therapy assessment is a widely accepted dynamic risk stratification tool among postoperative follow-up patients who have received TT (18, 19). In this study, we utilized the tool immediately after surgery to assist in the decision-making of RAI therapy in patients who underwent TT (20).

## Method

### Patients

A prospective single-center, observational study was conducted by recruiting patients who underwent TT from January 1^st^, 2016, to April 30^th^, 2022, at the Peking Union Medical College Hospital. The study included adult DTC patients who did not receive RAI therapy after TT and cervical LN dissection, with capsule invasion and/or LNM, confirmed by postoperative pathology, and with excellent or indeterminate response in the immediate postoperative response to therapy assessment (21). Patients with local recurrence or DM, incomplete clinical information, or receiving RAI were excluded. This study adhered to the guidelines of the Declaration of Helsinki and was approved by the Ethics Review Committee of Peking Union Medical College Hospital. All the patients signed informed consent to participate in the study.

### Procedures

In this study, TT with central LN dissection (CLND) was undergone in patients with cN1 or bilateral cancer or primary tumor size over 2cm (12, 21). For cN1b patients, based on preoperative examination (ultrasound, computer tomography scan, and/or fine-needle aspiration biopsy), a lateral LN dissection (LLND) covering level IIa-IV was performed. Levels I, IIb, and V; were not routinely dissected unless LN metastases were confirmed. Before discharge, the baseline information was collected, including age, gender, primary tumor size, number of lesions and metastatic LN, histological subtype, BRAF mutation, etc.

### Follow-up protocol and endpoints

The immediate postoperative response to therapy assessment was performed 1.5-2 months after surgery. All the accessed patients required four weeks of levothyroxine withdrawal before the assessment to ensure a significant serum thyroid-stimulating hormone (TSH) ≥30μIU/ml level (4). Patients were also instructed to avoid iodine-rich foods and iodine-containing drugs during this period.

The included patients were subjected to postoperative routine follow-up of thyroid cancer at three months, six months, and 12 months, followed by every six months within two years and annually over two years. Thyroglobulin (Tg), thyroglobulin antibodies (TgAb), and TSH were evaluated, and an ultrasound was performed at each follow-up visit. Patients who did not return to follow-up on time were contacted through telephone or social media to obtain the results of relevant laboratory and imaging investigations.

The primary endpoint of this study was DFS, which was defined as the time from the primary surgery to recurrence or death. The secondary endpoint was the classification of response to therapy assessment at the last follow-up, subdivided into four categories: excellent response, indeterminate response, biochemical incomplete response, and structural incomplete response, based on the 2015 American Thyroid Association (ATA) guidelines. For TgAb-positive patients with negative imaging, those with declining or stable TgAb (less than a 50% rise from the initial value) were classified as an indeterminate response, and those with a rising TgAb trend (a >50% rise from the initial value) were designated as biochemical incomplete response (22, 23).

### Statistical analysis

All the data analyses were performed using R version 4.1.2. Descriptive statistics were used to summarize the data. The continuous variables were expressed as mean ± standard deviation or median (range), and the categorical variables were expressed as numbers and percentages. The groups were compared and analyzed appropriately using the *Student’s* t-test, Kruskal-Wallis test, Mann-Whitney U test, or χ2 test. DFS was estimated by the Kaplan-Meier method. The statistical significance was defined at a p-value < 0.05.

## Results

**Figure 1:**
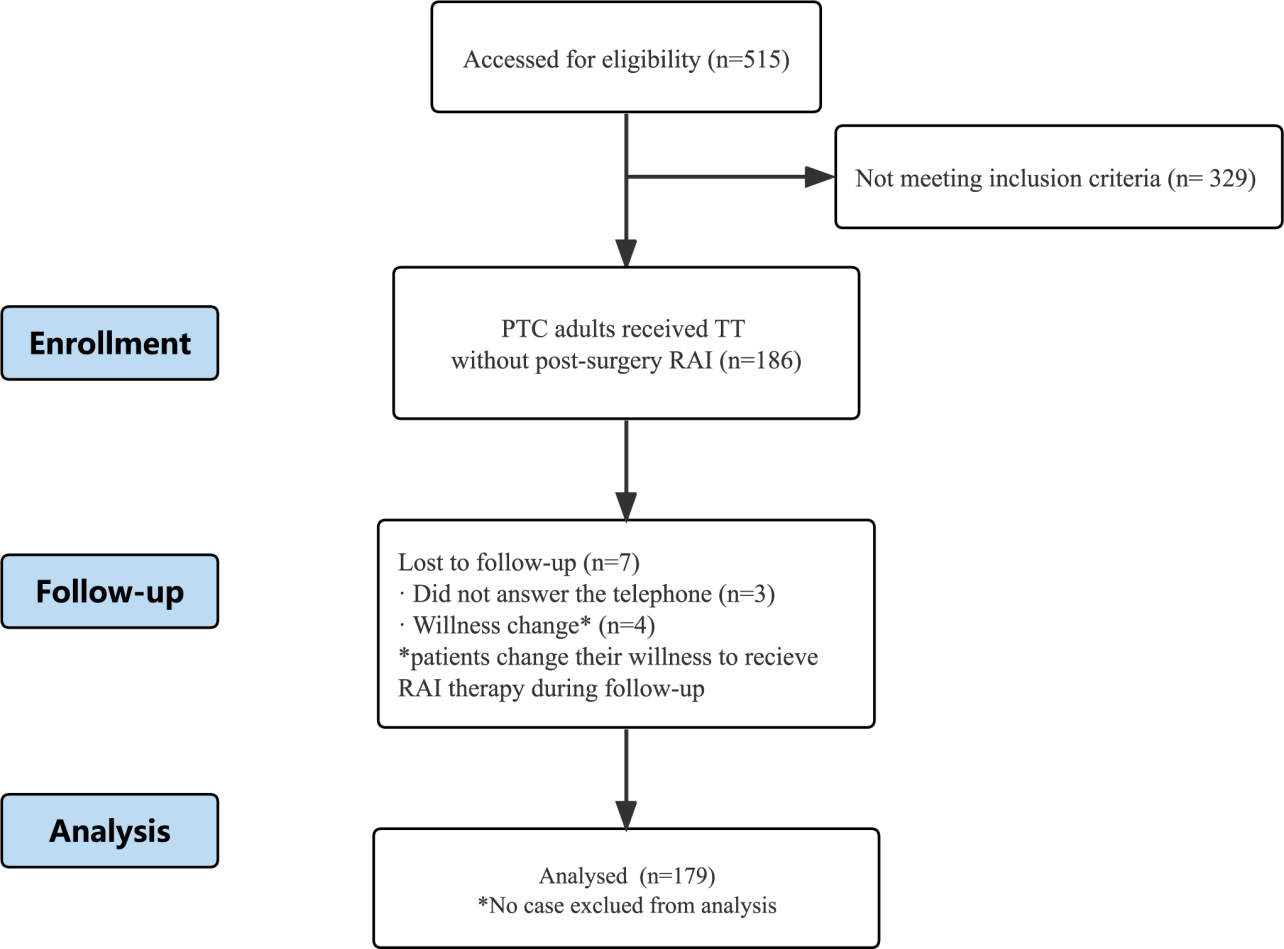
Flow diagram for inclusion and follow-up of this study.

Out of the 515 patients evaluated, 186 patients satisfied the inclusion criteria. Three patients were lost to follow-up, and four changed their willingness to receive RAI therapy (**Figure 1**). Finally, 179 patients were included in the study. The average follow-up period was 29.85 ± 17.44 months with a median of 26.40 months (range:4.47-69.23 months).

### Clinical characteristics

**Table 1** summarizes the baseline information of the included patients. The mean age was 44.6 ± 10.1 years; 74.9% were females. The average tumor size was 1.24 ± 0.66 cm. LNM suggested by the preoperative US was found in 74 (41.3%) patients. Among them, 48.6% had lateral LNM and received LLND. The number of harvested LN during surgery for all patients, patients who received CLND only, and patients who received both CLND and LLND was 21.2, 15.1, and 45.4 on average, respectively. Pathologically confirmed LNM was present in 106 (59.2%) patients, and 26.4% had over five involved LN. Chronic lymphocytic thyroiditis was found in 71 (39.7%) patients. Tumor invasion of the capsule and/or surrounding tissue was present in 157 (87.7%) patients. Twenty-four patients had minor extrathyroidal extension (mETE), and three had important structures involved but achieved R0 dissection.

**Table 1:** Demographic and clinicopathologic characteristics of included patients.

		Total (N=179)
Gender		
Female		134 (74.9%)
Male		45 (25.1%)
Age		44.6 ± 10.1
Preoperative TSH (μIU/mL)		2.16 ± 2.61
Preoperative Tg (ng/mL)		23.1 ± 48.9
Preoperative TgAb (IU/ml)		158 ± 460
LNM (by US)		
No		105 (58.7%)
Yes		74 (41.3%)
Without lateral LNM		38 (21.2%)
With lateral LNM		36 (20.1%)
Metastatic lymph node size (cm, N=74)		0.49 ± 0.71
Lateral lymph node dissection		
No		143 (79.9%)
Yes		36 (20.1%)
Primary tumor size (CM)		1.24 ± 0.66
Pathological subtype		
Papillary		126 (70.4%)
Follicular		28 (15.6%)
Papillary and follicular		22 (12.3%)
Others		3 (1.7%)
Multifocality		
No		79 (44.1%)
Yes		100 (55.9%)
Tumor invasion		
No		22 (12.3%)
Capsular invasion		130 (72.6%)
Extracapsular extension		27 (15.1%)
Chronic lymphocytic thyroiditis		
No		108 (60.3%)
Yes		71 (39.7%)
Number of LN harvested		
Total		21.2 ± 16.7
CLND only		15.1 ± 9.5
CLND+LLND		45.4 ± 17.4
LNM (by postoperative pathology)		
No		73 (40.8%)
Yes		106 (59.2%)
≤ 5 LN involved		78 (43.6%)
> 5 LN involved		28 (15.6%)
ATA risk stratification		
Low risk		110 (61.5%)
Intermediate risk		66 (36.9%)
High risk		3 (1.7%)
BRAF mutation (N=97)		
No		16 (16.5%)
Yes		81 (83.5%)

TSH, thyroid-stimulating hormone; Tg, thyroglobulin; TgAb, thyroglobulin antibody; LNM, lymph node metastases; US, ultrasound; LN, lymph node; CLND, center lymph node dissection; LLND, lateral lymph node dissection.

### Immediate postoperative response to therapy assessment

**Table 2** shows the outcomes of the immediate postoperative response to therapy assessment. Thirty-three patients were found TgAb positive (115 IU/mL). Of those, 75.8% had lower TgAb than pre-operation, and others had stable TgAb. All patients had stimulated Tg (s-Tg) <10ng/ml. In addition, the s-Tg in TgAb-negative patients were 69.9% <1ng/ml, 87.0% <2ng/ml, 98.0% <5ng/ml. Of note, no abnormal iodine uptake was found in 99 patients who underwent a diagnostic whole-body scan (DX-WBS), and 16.2% of them had no residual thyroid bed uptake. Excellent and indeterminate responses were 36.3% and 63.7% in the immediate postoperative assessment, respectively. Three cases involved important structures, one was classified as an excellent response, and two were indeterminate.

**Table 2:** The results of immediate postoperative response to therapy assessment.

	Total (N=179)	
	TgAb negative (N=146)	TgAb positive (N=33)
s-Tg level (ng/ml)	0.89 ± 1.14	0.40 ± 0.86
Distribution of s-Tg		
<0.2ng/ml	47 (32.2%)	22 (66.7%)
0.2-1ng/ml	55 (37.7%)	8 (24.2%)
1-2ng/ml	25 (17.1%)	2 (6.1%)
2-5ng/ml	16 (11.0%)	1 (3.0%)
5-10ng/ml	3 (2.1%)	0%
TgAb level change		
Decrease	–	25 (75.8%)
Stable	–	8 (24.2%)
Abnormal US (No)	146 (100%)	33 (100%)
DX-WBS		
NA*	64 (43.8%)	16 (48.5%)
Residual thyroid bed uptake	67 (45.9%)	16 (48.5%)
No uptake	15 (10.3%)	1 (3.0%)
Immediate postoperative response		
Excellent	65 (44.5%)	0%
Indeterminate	81 (55.5%)	33 (100%)

*NA, not available; s-Tg, stimulated thyroglobulin; DX-WBS, diagnostic whole-body scan.

### Primary and secondary endpoints

**Figure 2:**
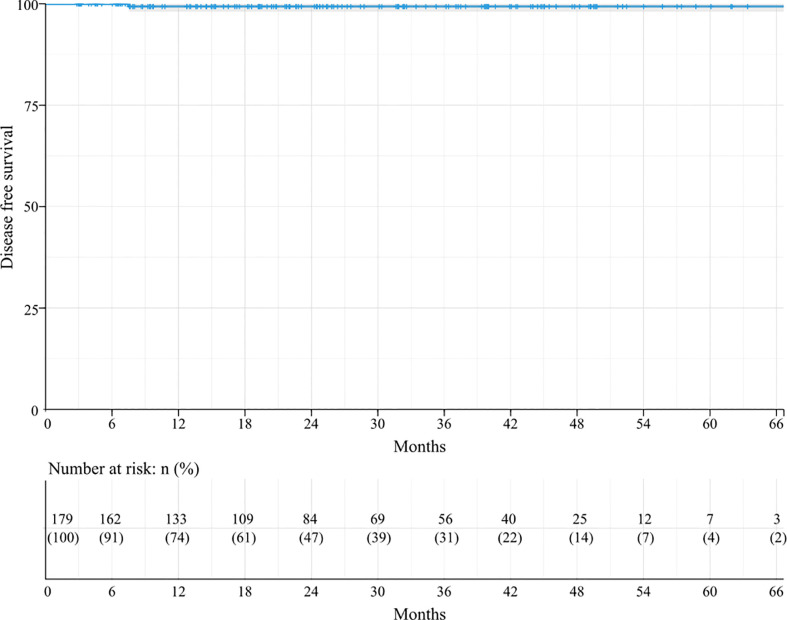
Kaplan-Meier survival curve for DFS.

The Kaplan–Meier analysis for DFS revealed that the 3-year and 5-year DFS rates in these 179 patients were 99.3%, and the median DFS was not achieved (**Figure 2**). DFS did not differ among the subgroups in all the factors.

Seven patients were TgAb positive during the last follow-up. Of these, one had elevated TgAb, and six had reduced TgAb. **Table 3** summarizes the unstimulated Tg (u-Tg) of 172 TgAb-negative patients at the last follow-up. The average u-Tg was 0.058± 0.058ng/ml. All the patients had u-Tg ≤ 1ng/ml, and 96.5% were <0.2ng/ml.

**Table 3:** u-Tg level and distribution at the last follow-up.

	u-Tg level (ng/ml)		u-Tg distribution			
		P value	<0.2 ng/ml	0.2-1 ng/ml	>1 ng/ml	P value
Total (N=172)	0.058 ± 0.058		166 (96.5%)	6 (3.5%)	0%	–
Gender						
Female (N=129)	0.050 ± 0.032	0.012	127 (98.4%)	2 (1.6%)	0%	0.035
Male (N=43)	0.085 ± 0.098		39 (90.7%)	4 (9.3%)	0%	
Age						
<55 (N=149)	0.060 ± 0.062	0.922	134 (96.4%)	5 (3.6%)	0%	>0.999
≥55 (N=33)	0.054 ± 0.039		35 (97.2%)	1 (2.8%)	0%	
Lateral LN dissection						
No (N=137)	0.058 ± 0.057	0.762	132 (96.4%)	5 (3.6%)	0%	>0.999
Yes (N= 35)	0.061 ± 0.061		34 (97.1%)	1 (2.9%)	0%	
Pathological subtype						
PTC (N=119)	0.063 ± 0.067	0.674	113 (95.0%)	6 (5.0%)	0%	0.574
FTC (N= 28)	0.044 ± 0.011		28 (100%)	0%	0%	
PTC and FTC (N=22)	0.054 ± 0.035		22 (100%)	0%	0%	
Others (N= 3)	0.040 ± 0		3 (100%)	0%	0%	
Multifocality						
No (N= 74)	0.056 ± 0.049	0.727	72 (97.3%)	2 (2.7%)	0%	0.701
Yes (N= 98)	0.060 ± 0.064		94 (95.9%)	4 (4.1%)	0%	
Tumor invasion						
No (N=20)	0.051 ± 0.031	0.013	20 (100%)	0%	0%	0.087
Capsular invasion (N=125)	0.051 ± 0.042		122 (97.6%)	3 (2.4%)	0%	
Extracapsular extension (N=27)	0.096 ± 0.105		24 (88.9%)	3 (11.1%)	0%	
Tumor size						
≤1cm (N=73)	0.063 ± 0.061	0.183	69 (94.5%)	4 (5.5%)	0%	0.403
>1cm (N= 99)	0.055 ± 0.055		97 (98.0%)	2 (2.0%)	0%	
LNM						
No (N=70)	0.052 ± 0.044	0.211	69 (98.6%)	1 (1.4%)	0%	0.265
≤5 LN involved (N=75)	0.059 ± 0.063		72 (96.0%)	3 (4.0%)	0%	
>5 LN involved (N=27)	0.074 ± 0.074		25 (92.6%)	2 (7.4%)	0%	
s-Tg level (N=146)*						
1ng/ml as cutoff value						
≤1ng/ml (N=102)	0.047 ± 0.024	<0.001	102 (100%)	0%	0%	<0.001
>1ng/ml (N=44)	0.095 ± 0.100		38 (86.4%)	6 (13.6%)	0%	
2ng/ml as cutoff value						
≤2ng/ml (N=126)	0.052 ± 0.045	<0.001	124 (98.4%)	2 (1.6%)	0%	<0.001
>2ng/ml (N=20)	0.122 ± 0.108		16 (80.0%)	4 (20.0%)	0%	
5ng/ml as cutoff value						
≤5ng/ml (N=142)	0.060 ± 0.060	0.143	137 (96.5%)	5 (3.5%)	0%	0.180
>5ng/ml (N=4)	0.105 ± 0.105		3 (75.0%)	1 (25.0%)	0%	
Immediate postoperative response						
Excellent (N=65)	0.047 ± 0.024	0.062	65 (100%)	0%	0%	0.090
Indeterminate (N=117)	0. 064 ± 0.068		111 (94.8%)	6 (5.2%)	0%	
BRAF mutation						
No (N=15)	0.043 ± 0.008	0.460	15 (100%)	0%	0%	>0.999
Yes (N=80)	0.062 ± 0.064		76 (95.0%)	4 (5.0%)	0%	

*s-Tg level: The s-Tg level measured at the immediate postoperative response to therapy assessment and only include the TgAb-negative patients.

u-Tg, unstimulated thyroglobulin.

During the last follow-up, 92.2% of patients achieved excellent responses (**Table 4**), and only one patient had LN recurrence. Females had a significantly better response distribution than males (P =0.023). The proportions of excellent responses were 95.5% and 84.4% for the two groups, respectively. Among the TgAb negative patients, significant differences were found in response distribution between the groups when the cutoff value of s-Tg was either 1ng/ml or 2ng/ml (p<0.001). The proportions of excellent responses were 100% and 84.1% for over and under 1ng/ml group, respectively. Additionally, 98.4% of the under 2ng/ml group and 75.0% of the over 2ng/ml group were classified as excellent responses. Moreover, 98.5% of excellent response patients and 88.6% of indeterminate response patients at the immediate postoperative assessment achieved excellent response at the last follow-up, with significant differences between the groups (P=0.004). No significant difference was observed between the groups regarding age, lateral LN dissection, pathological subtype, multifocality, LNM, tumor invasion, tumor size, and BRAF mutation (P>0.05).

**Table 4:** Response to therapy at the last follow-up.

	Distribution of response				
	Excellent	Indeterminate	Biochemical incomplete	Structural incomplete	P value
Total (N=179)	165 (92.2%)	12 (6.7%)	1 (0.6%)	1 (0.6%)	
Gender					
Female (N=134)	128 (95.5%)	5 (3.7%)	0%	1 (0.7%)	0.023
Male (N=45)	38 (84.4%)	6 (13.3%)	1 (2.2%)	0%	
Age					
<55 (N=145)	133 (91.72%)	10 (6.9%)	1 (0.7%)	1 (0.7%)	>0.99
≥55 (N=34)	32 (94.1%)	2 (5.8%)	0%	0%	
Lateral LN dissection					
No (N=143)	132 (92.3%)	10 (7.0%)	0%	1 (0.7%)	0.533
Yes (N= 36)	33 (91.7%)	2 (5.6%)	0%	1 (2.8%)	
Pathological subtype					
PTC (N=126)	115 (91.3%)	10 (8.0%)	1 (0.8%)	0%	0.408
FTC (N= 28)	26 (92.9%)	2 (7.1%)	0%	0%	
PTC and FTC (N=22)	21 (95.5%)	0%	0%	1 (4.5%)	
Others (N= 3)	3 (100%)	0%	0%	0%	
Multifocality					
No (N= 79)	73 (92.4%)	5 (6.3%)	1 (1.3%)	0%	0.861
Yes (N= 100)	92 (92.0%)	7 (7.0%)	0%	1 (1.0%)	
Tumor invasion					
No (N=22)	20 (90.9%)	1 (4.5%)	1 (4.5%)	0%	0.322
Capsular invasion (N=130)	121 (93.1%)	8 (6.2%)	0%	1 (0.8%)	
Extracapsular extension (N=27)	24 (88.9%)	3 (11.1%)	0%	0%	
Tumor size					
≤1cm (N=75)	68 (90.7%)	6 (8.0%)	0%	1 (1.3%)	0.571
>1cm (N= 104)	97 (93.3%)	6 (5.8%)	1 (1.0%)	0%	
LNM					
No (N=73)	70 (95.9%)	3 (4.1%)	0%	0%	0.233
≤5 LN involved (N=78)	71 (91.0%)	6 (7.7%)	0%	1 (1.3%)	
>5 LN involved (N=28)	24 (85.7%)	3 (10.7%)	1 (3.6%)	0%	
s-Tg level (N=146)					
1ng/ml as cutoff value					
≤1ng/ml (N=102)	102 (100%)	0%	0%	0%	<0.001
>1ng/ml (N=44)	37 (84.1%)	6 (13.6%)	0%	1 (2.3%)	
2ng/ml as cutoff value					
≤2ng/ml (N=126)	124 (98.4%)	2 (1.6%)	0%	0%	<0.001
>2ng/ml (N=20)	15 (75.0%)	4 (20.0%)	0%	1 (5.0%)	
5ng/ml as cutoff value					
≤5ng/ml (N=142)	136 (95.8%)	5 (3.5%)	0%	1 (0.7%)	0.180
>5ng/ml (N=4)	3 (75.0%)	1 (25.0%)	0%	0%	
Immediate postoperative response					
Excellent (N=65)	64 (98. 5%)	0%	1 (1.5%)	0%	0.004
Indeterminate (N=114)	101 (88.6%)	12(10.5%)	0%	1 (0.9%)	
BRAF mutation					
No (N=16)	14 (87.5%)	1 (6.3%)	1 (6.3%)	0%	0.322
Yes (N=81)	73 (90.1%)	7 (8.6%)	0%	1 (1.2%)	

## Discussion

RAI therapy is an effective postoperative intervention for a significant proportion of DTC patients, especially those with severe local invasion or DM. However, the beneficial effect of RAI therapy in patients with capsular invasion and/or LNM remains controversial (4). Several studies have postulated that RAI therapy is unnecessary for patients receiving an ideal surgery. Ballal et al. reported a 100% 10-year OS for intermediate-risk patients with or without RAI therapy after surgery and a 92% and 90% DFS rate for the two groups, respectively (16). Kim et al. suggested that RAI therapy does not decrease the LRR even in intermediate-risk patients with ETE (HR=0.762, P=0.228), LNM (HR=0.804, P=0.349), or multifocal (HR=0.103, P=0.926) (17). Similar results were obtained in the current study. The 5-year DFS reached 100% for both groups (data not shown) among patients with excellent response and TgAb-negative patients with s-Tg<2ng/ml and an indeterminate response in the immediate postoperative assessment. Moreover, the proportions of excellent responses at the last follow-up were 98.5% and 98.4%, respectively.

Some postoperative DTC patients with recurrence risk features could still have a good outcome without RAI therapy. A practical question is how to screen out this particular group of patients. To the best of our knowledge, no study is yet to answer this question. In the present study, we applied response to therapy assessment immediately after surgery to help screen out these patients. Our results revealed no recurrence in all the patients (with s-Tg<1ng/ml) who had an excellent response in the immediate postoperative assessment. Similarly, Ibrahimpasic et al. reported no significant difference in the 5-year recurrence-free survival (RFS) between the group of intermediate-risk patients with and without RAI therapy who have s-Tg<1ng/ml (97% vs. 96%, P=0.234) (24). Moreover, no recurrence was observed in 126 TgAb-negative patients with s-Tg<2ng/ml. Among them, 124 (98.4%) patients achieved excellent responses during the last follow-up. Similarly, Rosario et al. observed a very low recurrence rate (3.5%) in intermediate-risk patients with s-Tg<2ng/ml (25). Therefore, we consider RAI therapy might be unnecessary for the scenarios mentioned above. The preliminary results indicate that immediate postoperative assessment might be able to help avoid unnecessary RAI therapy among patients with risk recurrence features.

Although Momesso et al. designed response to treatment assessment for postoperative patients without RAI (26), we still incorporated the original version in this study. This is because the Tg cutoff values set for stratification were too lenient and the study population differs from the current study in the without RAI therapy version(91.5% low-risk patients) (20). Moreover, the clinical outcome of surgery alone is close to combined RAI therapy with the improvements in surgical technique and instruments (over 10% of patients in this study achieved negative iodine uptake in postoperative DX-WBS). Therefore, assessment with stringent standards can better evaluate the disease status of patients during the immediate postoperative period and assist the RAI treatment decision-making.

Postoperative LNM is a significant concern among surgeons and patients with an incidence of 20%-50% (4, 27). RAI therapy is considered an efficient method to treat LNM. However, lymph node recurrence is not rare despite RAI treatment. Furthermore, a long-term study from Mayo Clinic shows no difference in tumor recurrence between the TT and TT+RAI groups in both patients with and without LNM (28). Thus, the role of RAI therapy in LNM treatment remains doubtful. In the present study, only one patient was found with lateral LNM at seven months postoperatively but achieved excellent response after second surgery (without RAI therapy). The rates of excellent response during the last follow-up of patients with LNM (N=106/179), less (N=78/179), and over five LN involved (N=28/179) were 89.6%, 91.0%, and 85.7%, respectively, much better than the results reported by Leboulleux et al. in low-risk patients (73.0% excellent response with only 44% patients receiving neck dissection) (22). Moreover, the 5-year DFS estimates were 99.3% and 100% for all the patients and patients with over five LN involved, respectively, better than the result reported by Kim et al. in intermediate-risk patients without RAI treatment (a 5-year RFS of 95.7% with 83.9% patients received neck dissection) (17). One possibility is that all patients in this study routinely underwent cervical LN dissection, decreasing the risk of recurrence (29–31). Thus, thorough resection in surgery could avoid RAI therapy, and LNM or even over five LN involved should not be the basis of a routine RAI therapy.

A minor extrathyroidal extension is no longer an indicator of intermediate-risk patients in the eighth TNM staging system. Therefore, a part of patients with mETE will be downstaged and did not receive RAI (32). Moreover, a study by Rosario et al. reported that structure recurrence occurred only in 2.2% of patients with mETE and treated without RAI (33). Similarly, this study observed no recurrence in 24 patients with mETE, and 87.5% of these patients achieved excellent response at the last follow-up. Therefore, patients with mETE might not require ablation with RAI. Of note, all the three patients involved with important structures and R0 resection achieved excellent responses during the last follow-up. In these patients, evaluating their disease status for RAI therapy decisions still needs further exploration.

Endogenous TgAb may interfere in Tg measurement, impairing its ability to reflect the condition of the patients. Therefore, monitoring postoperative conditions in TgAb-positive patients can be challenging (4, 34). Previous studies have shown that the changing trends of TgAb can help the postoperative monitoring. A decreasing trend of TgAb could indicate remission (35), and a > 50% rise of TgAb could indicate a high risk of recurrence (23). In this study, most of the TgAb-positive patients had a decreasing trend of TgAb and showed excellent response during the last follow-up. Only one patient with a rising trend was assessed as biochemical incomplete and was not treated with RAI since no recurrence or metastatic lesion was indicated in the DX-WBS. Therefore, it is feasible for TgAb-positive patients with a stable or declining trend of TgAb to not receive RAI therapy.

Post-treatment whole body scans (ptWBS) are currently recommended in patients receiving RAI therapy. However, the accuracy of DX-WBS for identification of metastasis is controversial (36, 37). Nava et al. reported a sensitivity of 29% and a specificity of 97% for DX-WBS in ATA low-intermediate-risk DTC patients, indicating it should be reconsidered for routine DX-WBS in this subgroup (38). Even so, we still recommend that it should be routinely performed in the immediate postoperative assessment for the patient without RAI therapy. The DX-WBS can provide additional information for TgAb-positive patients since the trend of TgAb cannot monitor the condition precisely. Additionally, monitoring of disease condition by Tg alone, even in Tg-Ab negative patients, could still miss the local recurrence or DM (39).

Previous studies postulate a worse prognosis in male DTC patients than in females (40). In the present study, though there was no difference in recurrence between the male and female patients, male patients depicted a worse distribution of response to therapy at the last follow-up. In contrast to gender, no difference in recurrence and response distribution was observed between the BRAF mutate. Unmutated patients in this study were in line with the latest randomized controlled study (RCT) by Leboulleux et al. in low-risk patients (22), indicating that the presence of BRAF mutation might not be an indication for RAI therapy.

For the first time, the current study applies the response to therapy assessment immediately after surgery in DTC patients to assist in decision making regarding RAI therapy, which broadens the utilization of existing assessment tools and helps avoid unnecessary RAI treatment. Of note, the results of this study are based on observations on only 179 patients over a short period of time and should only applied on the selected low-intermediate risk DTC patients. For intermediate-risk patients, though not uniform, there is a considerable body of evidence in favor of RAI therapy among them (41, 42). For example, Ruel et al. reported a 29% reduction in the risk of death among 15,418 intermediated patients received RAI, compared with 6,452 patients without RAI (43). However, according to our data, intermediate-risk patients underwent thorough resection in surgery and with an excellent or indeterminate response in the immediate postoperative assessment can still have an excellent short-term prognosis without RAI. In these patients, RAI therapy should be considered more cautiously considering the risks and benefits.

Several factors limited this study. First, the current study was a single-center and single-arm investigation with a relatively small sample size, which limits the strength of the evidence; multicenter validation and controlled studies with larger number of patients are warranted. Second, an follow-up period of two and a half years on average was relatively short, and some recurrence may not have had time to be observed. Although several previous studies reported that most recurrence occurred generally in the first 5 years of follow-up in patients underwent RAI therapy (44, 45), a high risk of late recurrence was also reported in young patients without RAI therapy (46). Therefore, a more extended follow-up period is required to assess recurrence.

## Conclusion

The current study explored the criteria to avoid unnecessary RAI therapy in postoperative DTC patients with capsular invasion and/or LNM. It was observed that the immediate postoperative response to therapy assessment was an essential basis. For TgAb-negative patients with excellent response, indeterminate response, s-Tg<2ng/ml in the immediate postoperative assessment and TgAb-positive patients with a decrease or stable trend of TgAb, RAI therapy might be unnecessary.

## Data availability statement

The raw data supporting the conclusions of this article will be made available by the authors, without undue reservation.

## Ethics statement

The studies involving human participants were reviewed and approved by Ethics Review Committee of Peking Union Medical College Hospital. The patients/participants provided their written informed consent to participate in this study.

## Author contributions

Protocol and design of study: HZ, C-HL, X-YL. Acquisition and/or management of data: HZ, C-H.L, YZ. Analysis and/or interpretation of data: HZ, CH, X-YL. Drafting and revising the manuscript: HZ, X-YL, L-YZ, XZ, YX, Y-SL, Y-WL, H-FL. All authors contributed to the article and approved the submitted version.

## Acknowledgments

The authors would like to thank Yanlong Li, Ph.D., for statistical consultation during data collection and analysis, and MogoEdit (https://www.mogoedit.com) for its English editing during the preparation of this manuscript.

## Conflict of interest

The authors declare that the research was conducted in the absence of any commercial or financial relationships that could be construed as a potential conflict of interest.

## Publisher’s note

All claims expressed in this article are solely those of the authors and do not necessarily represent those of their affiliated organizations, or those of the publisher, the editors and the reviewers. Any product that may be evaluated in this article, or claim that may be made by its manufacturer, is not guaranteed or endorsed by the publisher.
